# Sex impacts Th1 cells, Tregs, and DCs in both intestinal and systemic immunity in a mouse strain and location-dependent manner

**DOI:** 10.1186/s13293-016-0075-9

**Published:** 2016-04-05

**Authors:** Marlies Elderman, Adriaan van Beek, Eelke Brandsma, Bart de Haan, Huub Savelkoul, Paul de Vos, Marijke Faas

**Affiliations:** Top Institute Food and Nutrition, Wageningen, The Netherlands; Division of Medical Biology, Department of Pathology and Medical Biology, University Medical Centre Groningen, Groningen, The Netherlands; Cell Biology and Immunology Group, Wageningen University, Wageningen, The Netherlands; Department of Obstetrics and Gynaecology, University of Groningen and University Medical Centre Groningen, Groningen, The Netherlands

**Keywords:** Sex differences, Peyer’s patches, Intestinal immune cells, T cells, Dendritic cells, Macrophages, Natural killer cells, C57Bl/6, BALB/c

## Abstract

**Background:**

Males and females have a different predisposition for the development of intestinal disorders, like inflammatory bowel disease (IBD). We hypothesized that sex specific differences in intestinal immune responses may underlie this bias. To test this hypothesis, we studied sex differences in immune cell populations in the Peyer’s patches (PP). For comparison with systemic immunity, we studied spleen cells.

**Methods:**

Two mouse strains with different susceptibility for developing colitis (BALB/c and C57Bl/6) were used. Using flow cytometry, we measured the percentage of T cells, Th1, Th17, and Treg cells in the PP and spleen. In addition, we measured the percentages of NK cells, macrophages, myeloid, and lymphoid dendritic cells (DCs) and their expression of CD80 and CD103. Moreover, we measured percentages of monocyte subsets in the peripheral circulation. Results were tested using two-way ANOVA, *p* < 0.05.

**Results:**

Males had a lower percentage of T cells in both PP and spleen (PP BALB/c 22.1 %, B6 13.6 %; spleen BALB/c 4.7 %, B6 19.9 %) but a higher percentage of Th1 cell in both tissues (PP BALB/c 350 %, B6 109.5 %; spleen BALB/c 48.7 %, B6 41.9 %) than females. They also had a higher percentage of Tregs in the spleen than females (BALB/c 20.5 %, B6 4.5 %). Furthermore, males had a higher percentage of CD80^+^ DCs in both the PP and spleen (lymphoid DCs in PP BALB/c 104.7 %, B6 29.6 %; spleen BALB/c 72.2 %, B6 44.2 %; myeloid DCs in PP BALB/c 80.5 %, B6 93.3 %; spleen BALB/c 88.5 %, B6 50.8 %) and a higher percentage of lymphoid CD103^+^ DCs in the spleen than females (BALB/c 41.5 %, B6 28.3 %). The percentage of NK cells was decreased in the spleen (BALB/c 12.5 %, B6 25.1 %) but increased in the PP (BALB/c 75.7 %, B6 78.6 %) of males as compared with females. Strain differences were also found in the PP; BALB/c mice had a higher percentage of T cells (males 58.1 %, females 75.5 %), a higher Th/Tc ratio (males 81.0 %, females 134.2 %), less FoxP3^+^CD25^−^ T cells (males 14.6 %, females 30.0 %), more DCs (males 14.8 %, females 15.7 %) and macrophages (males 67.9 %, females 141.2 %), and more NK cells (males 160 %, females164.3 %) than BALB/c mice.

**Conclusions:**

In this study, we show sex differences in intestinal and peripheral immune populations. These differences may underlie sex differences in intestinal disorders like IBD, and this information may be an important knowledge for the treatment of intestinal-related diseases.

## Background

Females have a higher risk of developing autoimmune diseases such as rheumatoid arthritis and systemic lupus erythematosus than males [1]. Sex differences in peripheral immune responses are thought to underlie this bias in autoimmune diseases [2]. Females generally react stronger and more vigorously with their adaptive immune response, whereas males have increased innate immune responses [3, 4]. Also, susceptibility to develop inflammatory bowel disease (IBD), such as Crohn’s disease, may be sex dependent; in European countries, females have a higher incidence of the disease, whereas in Asian Countries, men have a higher incidence [5, 6]. As IBD is characterized by chronic inflammation of the intestine [7], we hypothesized that sex differences in IBD may be due to sex differences in the intestinal immune response.

The intestinal immune system, also referred to as gut-associated lymphoid tissue (GALT), is in close contact with intestinal microbes and dietary antigens, making it distinct from the peripheral immune system [8]. The main challenge of the GALT is to distinguish harmless from harmful substances and to respond appropriately. The GALT consists of immune cells scattered through the lamina propria and organized lymph structures like the Peyer’s patches (PP) and mesenteric lymph nodes (MLN) [9]. Dendritic cells (DCs) in the PP expressing integrin sub-unit CD103 are able to differentiate T helper (Th) cells into FoxP3^+^ T regulatory cells (Tregs) [10]. Tregs can produce Il-10 and are important in controlling other T helper responses, preventing inflammation. Both CD103^+^ DCs and Tregs play an important role in intestinal homeostasis and tolerance and in the prevention of IBD [11, 12]. An intestinal immune response is induced by DCs and macrophages upon encountering an antigen. Depending on the type of antigen, DCs and macrophages induce the differentiation of T cells into effector T cells, such as T helper 1 (Th1), T helper 2 (Th2), and T helper 17 (Th17) cells, as well as the abovementioned Treg cells [13]. Other immune cells that play a role in intestinal homeostasis are natural killer (NK) cells. NKp46^+^ NK cells co-expressing transcription factor RORɣt in the intestine can produce interleukin 22 (IL-22) [14], which is involved in regulating mucosal barrier homeostasis and antimicrobial host defense [15]. Whether these intestinal immune cell subsets are sex dependent is still to be determined.

The aim of this study was therefore to test the hypothesis that sex affects intestinal immune cell populations. We focused on Th cell subsets, DCs, macrophages, and NK cells. In humans, susceptibility to develop IBD is related to genetic variation [16]. Therefore, sex differences in intestinal immune cells were researched in two mouse strains with different genetic backgrounds. The colitis-susceptible C57Bl/6 mice and the more resistant BALB/c mice were used [17, 18]. We used the PP as intestinal target tissue to determine sex differences, since it is an important place for immune sampling of antigens from the gut lumen [19]. As a reference for the peripheral immune system, we used the spleen. Male mice had a lower percentage of total T cells, but a higher percentage of Th1 than females in both organs. Furthermore, we found that the innate immune arm (DCs, macrophages, and NK cells) in the PP was enhanced in males as compared in females. This information may be an important knowledge for sex-dependent treatment of intestinal-related diseases.

## Methods

### Study design

This study was designed to assess the effect of sex on intestinal immune populations in mice. Two different mouse strains were used: C57B1/6OlaHsd (B6) and Balb/cOlaHsd (BALB/c) (*n* = 20 per strain). In both strains, two groups were present: female and male mice (*n* = 10 per group).

### Mice

Male and female wild-type B6 and BALB/c mice were purchased from Harlan (Harlan, Horst, The Netherlands) at an age of 8 weeks. Mice were housed in groups in isolated ventilated cages to limit environmental influences. Mice had ad libitum access to a D12450B diet (10 % fat) (Research Diets Services, Wijk bij Duurstede, The Netherlands) and water. All mouse experiments were performed after receiving approval of the Animal Care Committee of the Groningen University. Between an age of 3 and 5 months, all mice were sacrificed by cervical dislocation under anesthesia (isoflurane and oxygen). Subsequently their blood, spleen and PP were removed for further analysis. All female mice were sacrificed during the diestrus phase of their ovarian cycle to ensure low levels progesterone and estrogens.

### Isolation of spleen and Peyer’s patches cells

Single cell suspensions of spleens and PP were made by mechanical disruption of the tissues between two microscopy slides in 2-ml ice-cold RPMI containing 10 % (*v*/*v*) heat-inactivated fetal calf serum (FCS). Splenic red blood cells were eliminated by incubation with 4-ml ice-cold ammonium chloride. Part of the spleen suspensions (diluted in 2 ml RPMI + 10 % FCS (*v*/*v*)) were subsequently added to 3 ml of nycodenz gradient medium (NycoPrep™ 1.068 from Progen Biotechnik, Heidelberg, Germany) for enrichment of dendritic cells (DCs). Falcon tubes with cell strainer caps (Corning, Amsterdam, The Netherlands) (35 μm) were used to remove cell clumps before the cells were counted and used for staining.

### White blood cell isolation

Blood from the vena cava was collected in syringes containing EDTA (K2E, BD Biosciences, Breda, The Netherlands) to prevent clotting. The blood was incubated for 2 min with ice-cold ammonium chloride to eliminate red blood cells. After washing with ice-cold fluorescence-activated cell sorting (FACS) buffer (PBS + 10 % FCS (*v*/*v*) + 0.001 M EDTA), the suspensions were filtered with cell strainer caps to remove cell clumps before the cells were counted and used for staining.

### Cell staining

Spleen and PP cells were stained for T cell populations, DCs, macrophages, and natural killer (NK) cells, and blood was stained for monocytes. T cells were determined using CD3 and further subdivided into T cytotoxic (Tc) (CD8^+^) and T helper (Th) (CD8^−^) cells. Subsequently, Th cell subsets were determined using the markers Tbet (Th1), Gata3 (Th2), RORɣt (Th17), and FoxP3^+^CD25^+^ (Treg). Within the myeloid (MHC2^+^F4/80^-^CD11c^+^CD11b^+^) and lymphoid DCs (MHC2^+^F4/80^-^CD11c^+^CD11b^−^), expression of CD80 and CD103 was assessed. CD3 and NKp46 were used to mark natural killer (NK) cells. Monocytes (CD11b^+^Ly6G^−^) were stained for their expression of Ly6C, MHC2, and CD80. Antibody specifications are described in Table 1.

**Table 1:** Antibody specifications

Specificity	Clone name	Fluorochrome	Concentration (mg/ml)	Dilution^a^	Supplier
MHC2	2G9	Biotin	0.5	200×	BD Pharmingen ThermoFisher
		Streptavidin-Pacific Orange	1	100×	
F4/80	BM8	A700	0.5	75×	Biolegend
CD11c	N418	PE-Cy7	0.2	100×	Biolegend
CD11b	M1/70	APC-Cy7	0.2	200×	Biolegend
CD80	16-10A1	Pacific Blue	0.5	25×	Biolegend
CD103	2E7	PerCP-Cy5.5	0.2	25×	Biolegend
CD3	17A2	Pacific Blue	0.5	80×	Biolegend
CD8	53-6.7	A700	0.5	50×	Biolegend
CD25	PC61	PE-Cy7	0.2	50×	Biolegend
Tbet	eBio4B10	PE-Cy7	0.2	50×	eBioscience
Gata3	TWAJ	PerCP-Efluor710	0.2	50×	eBioscience
RORγt	B2D	PE	0.2	10×	eBioscience
FoxP3	FJK-16s	FitC	0.5	50×	eBioscience
NKp46	29A1.4	FitC	0.5	25×	Biolegend
Ly6G	1A8	Biotin	0.5	100×	Biolegend
		Streptavidin-Pacific Orange	1	100×	ThermoFisher
Ly6C	HK1.4	PE	0.2	300×	Biolegend
MHC2	M5/114.15.2	Pacific Blue	0.5	300×	Biolegend
CD80	16-10A1	PerCP-Cy5.5	0.2	50×	Biolegend

^a^Dilution used in a total volume of 25 μl supplemented with PBS + 10 % FCS

All antibodies were diluted in a volume of 25 μl, supplemented to a volume of 25 μl with FACS buffer (PBS + 10 % FCS (*v*/*v*)). Approximately 1 × 10^6^ spleen, PP, or white blood cells were incubated for 20 min in FACS buffer (10 % FCS (*v*/*v*)) containing 20 % (*v*/*v*) normal rat serum (Jackson, Newmarket, UK) and 2 % (*v*/*v*) Fc block (CD16/32) (Biolegend, Uithoorn, The Netherlands) to prevent non-specific antibody binding, followed by incubation in primary antibody mix for 30 min. The samples for detection of DCs and monocytes were stained with a biotinylated antibody (streptavidin-Pacific Orange) for 30 min. Subsequently, all samples were fixed in FACS lysing solution (BD Biosciences, Breda, The Netherlands) for 30 min. T and NK cell samples underwent intracellular staining and were washed twice with a permeabilization buffer (eBioscience, Vienna, Austria) after which they were incubated with an intracellular blocking medium (20 % (*v*/*v*) rat serum in permeabilization buffer) for 20 min. Next, these cells were incubated with a secondary antibody mix for 30 min. Washing was performed in between all incubation steps. The whole procedure was performed on ice and in the dark. Isotype control antibodies were used at the same concentration and purchased from the same company as the primary and secondary antibodies.

### Flow cytometry

Cell samples were analyzed using the LSR-II Flow Cytometer system (BD Biosciences, Breda, The Netherlands), using FACS Diva software. Analysis was performed using FlowJo version 10 software (FlowJo, LLC, Oregon, USA).

**Fig. 1:**
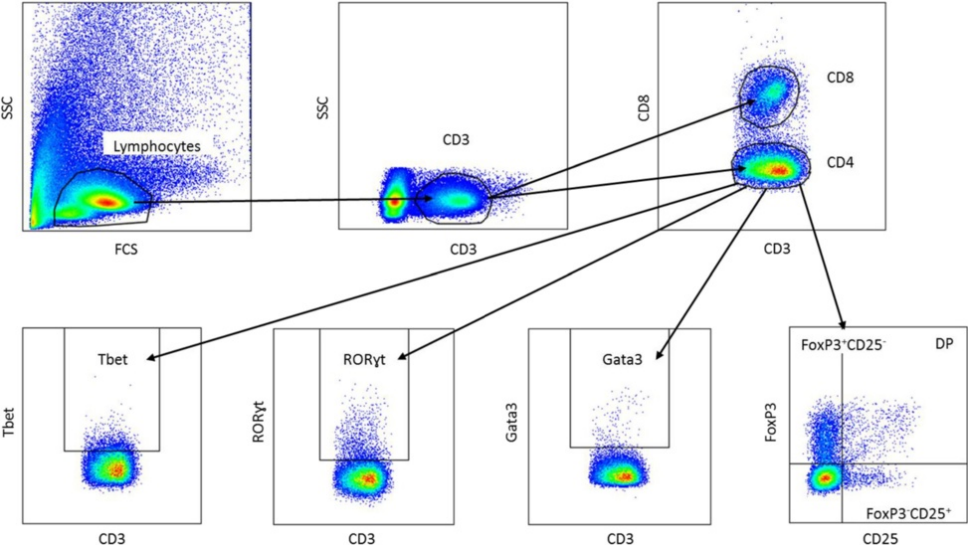
Gating strategy for the determination of T helper cells in the Peyer’s patches. Lymphocytes were gated on the bases of their size in the forward side scatter plot, and CD3^+^ T cells were selected. Next, CD4 and CD8 cells were selected by gating CD8 negative and positive cells, respectively. Within the CD8^−^ population, the expression of Tbet, Gata3, RORɣt, FoxP3, and CD25 were assessed. All isotype controls were set at 1 %

**Fig. 2:**
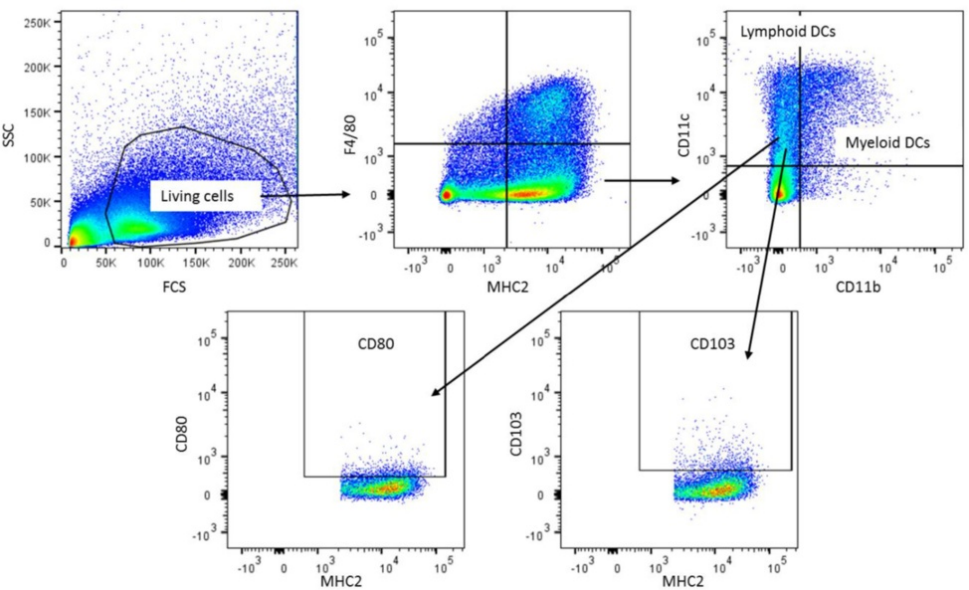
Gating strategy for the determination of dendritic cells and macrophages in the spleen. First living cells were selected based on size in the forward side scatter plot. Next MHC2^+^ and F4/80^+^ cells were selected as macrophages. Within the remaining MHC2^+^ and F4/80^−^ cells, myeloid DCs were gated as CD11c^+^CD11b^+^ and lymphoid DCs as CD11c^+^CD11b^−^. In both subsets, the expression of CD80 and CD103 was measured. All isotype controls were set at 1 %

**Fig. 3:**
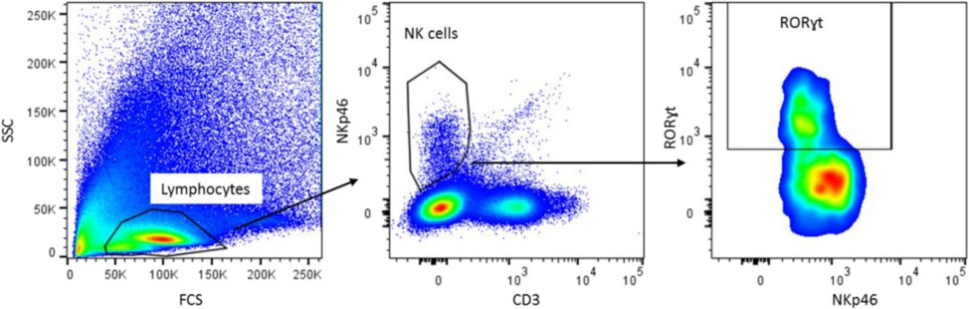
Gating strategy for the determination of natural killer cells in the Peyer’s patches. Lymphocytes were gated based on size in the forward side scatter plot. Within these cells, NK cells were determined by selecting CD3^-^NKp46^+^ cells. Within this subset the percentage of RORɣt^+^ cells was determined. All isotype controls were set at 1 %

**Fig. 4:**
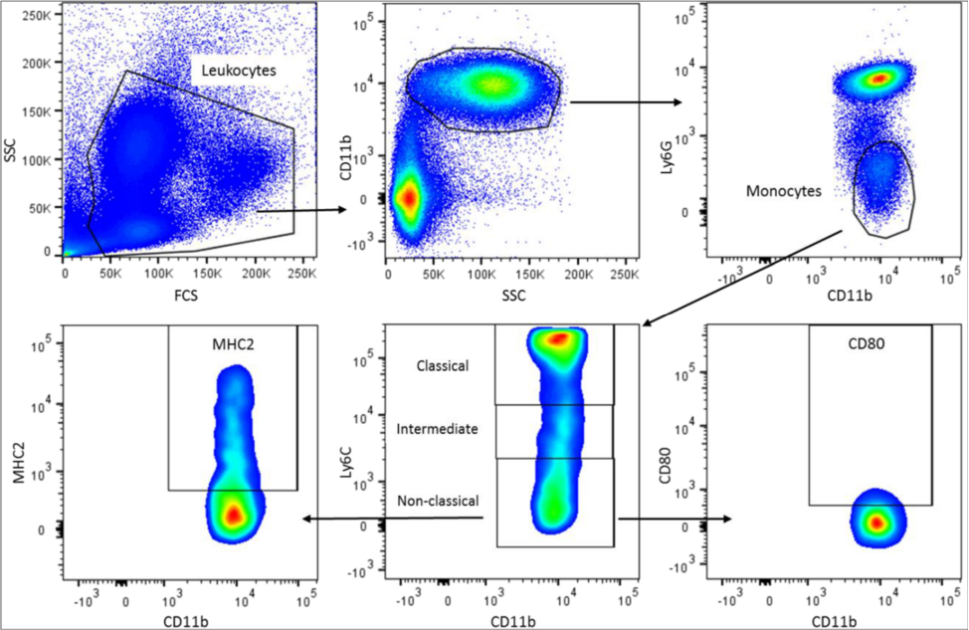
Gating strategy for the determination of monocytes in the blood. First, living cells were selected based on size in the forward side scatter plot. Next all CD11b^+^ cells were selected, and within this population, all Ly6G^−^ cells were gated to excluded granulocytes. Within the remaining monocytes, classical, intermediate, and non-classical monocytes were distinguished using ly6C. Within these three subsets MHC2^+^ and CD80^+^ cells were selected. All isotype controls were set at 1 %

The gating strategy for Th cells is shown in Fig. 1. Lymphocytes were gated based on the size in the forward side scatter plot, and T cells were determined by selecting CD3^+^ cells. Within the CD3^+^ cells CD8^+^ (Tc cells) and CD8^−^ (=CD4^+^ (Th cells)), cells were selected. The percentage of Tbet^+^, Gata3^+^, RORɣt^+^, FoxP3^+^ and CD25^+^ cells were assessed within the CD8^−^ cells. The gating strategy for DCs and macrophages is shown in Fig. 2. To gate DCs and macrophages, the first living cells were selected based on size in the forward side scatter plot. Next, MHC2^+^ and F4/80^+^ cells were selected as macrophages. Within the remaining MHC2^+^ and F4/80^−^ cells, myeloid DCs were gated as CD11c^+^CD11b^+^ and lymphoid DCs as CD11c^+^CD11b^−^. In both subsets, the expression of CD80 and CD103 was measured. The gating strategy for NK cells is shown in Fig. 3. To determine the percentage of NK cells, lymphocytes were gated based on the size in the forward side scatter plot. Within these cells, NK cells were determined by selecting CD3^-^NKp46^+^ cells. Within this subset, the percentage of RORɣt^+^ cells was determined. The gating strategy for monocytes is shown in Fig. 4. To gate the monocytes, the first living cells were selected based on the size in the forward side scatter plot. Next, all CD11b^+^ cells were selected, and within this population, all Ly6G^−^ cells were gated to excluded granulocytes. Within the remaining monocytes, classical, intermediate, and non-classical monocytes were distinguished using ly6C. Within these three subsets, MHC2^+^ and CD80^+^ cells were selected. All isotype controls were set at 1 %, and these gates were copied to all samples with the antibody mix.

### Statistics

All data are expressed as the mean with standard error of the mean (SEM). The Kolmogorov-Smirnov test was used to determine normal distribution of the data. The data were analyzed with a two-way ANOVA (TWA), which examines the influence of two different categorical independent variables (sex and strain) on one continuous dependent variable (immune cell population). This test thus allowed us to evaluate the overall effect of sex and gender on the immune cell populations. When the data were not normally distributed, a log transformation was performed before performing the two-way ANOVA. *p* values of 0.05 or smaller were considered statistically significant, and *p* values between 0.05 and 0.1 were defined as a trend.

## Results

### Effect of sex and strain on T helper cell subsets in the Peyer’s patches and spleen

**Fig. 5:**
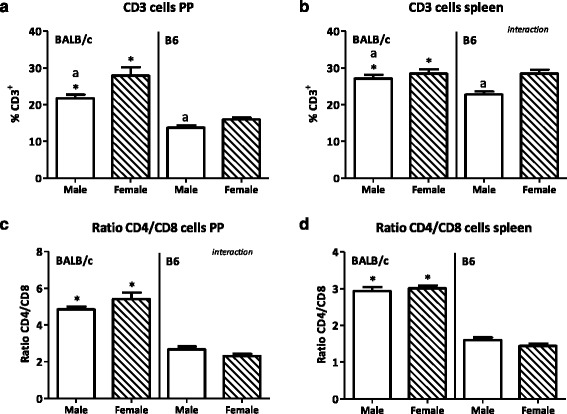
Effect of gender and mouse strain on T cells in the Peyer’s patches and spleen. Percentage of CD3^+^ T cells in Peyer’s patches (**a**) and spleen (**b**) of male, female, BALB/c, and B6 mice. The ratio of T helper cells/cytotoxic T cells within these T cells, in the Peyer’s patches (**c**) and spleen (**d**). Results are expressed as the mean + SEM and were tested using two-way ANOVA. Significant strain effects are indicated with an *asterisk* (*), and significant gender effects are indicated with the letter *a* in each graph (*p* < 0.05)

Males had a reduced percentage of T cells (CD3^+^) in the PP as compared with females (*p* < 0.01, Fig. 5a). However, sex did not affect the CD4^+^ Th/CD8^+^ cytotoxic T (Tc) cell ratio in the PP (Fig. 5c). In the spleen, we also found that males had a reduced percentage of T cells (*p* < 0.01, Fig. 5b), whereas the Th/Tc cell ratio in the spleen was similar between the two sexes (Fig. 5d). BALB/c and B6 mice had a different percentage of T cells in the PP. BALB/c mice had higher percentage of T cells than B6 mice (*p* < 0.001, Fig. 5a). Furthermore, BALB/c mice had a higher Th/Tc cell ratio than B6 mice (*p* < 0.001, Fig. 5c). Similar as in the PP, we found that BALB/c mice had a higher percentage of T cells and a higher Th/Tc cell ratio than B6 mice in the spleen (*p* < 0.05, Fig. 5b, d). Interaction between the effect of sex and strain was found in the percentage of T cells in the spleen and in the Th/Tc ratio in the PP.

**Fig. 6:**
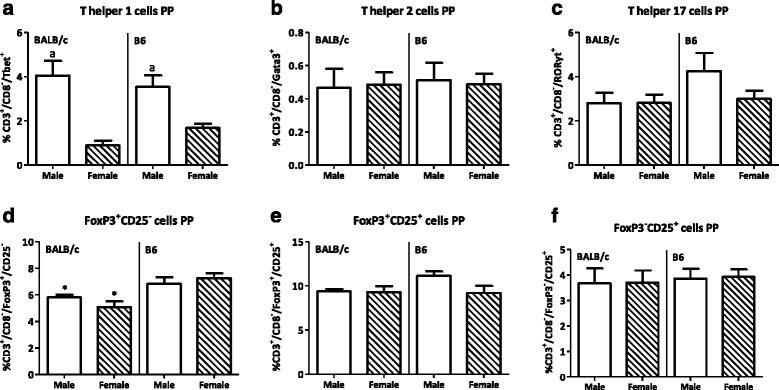
Effect of gender and mouse strain on T helper cell populations in the Peyer’s patches. Percentage of T helper 1 (**a**), T helper 2 (**b**), T helper 17 (**c**), FoxP3^+^CD25^-^ T regulatory cells (**d**), FoxP3^+^CD25^+^ T regulatory cells (**e**), and FoxP3^-^CD25^+^ effector T cells (**f**) in the Peyer’s patches of male, female, BALB/c, and B6 mice. T helper cells are expressed as the percentage of cells within the CD3^+^CD8^-^ population. Results are expressed as the mean + SEM and were tested using two-way ANOVA. Significant strain effects are indicated with an *asterisk* (*), and significant gender effects are indicated with the letter *a* in each graph (*p* < 0.05)

Th cell populations were subdivided into Th1 (Tbet^+^), Th2 (Gata3^+^), Th17 (RORɣt), and Treg (FoxP3^+^) cells. In the PP, males had an increased percentage of Th1 cells in the PP (*p* < 0.001, Fig. 6a); however, Th2 and Th17 cell percentages were similar between the two sexes (Fig. 6b, c). The percentage of FoxP3^+^CD25^-^ Tregs, FoxP3^+^CD25^+^ Tregs, and effector (FoxP3^-^CD25^+^) Th cells in the PP were similar between males and females (Fig. 6d–f). Strain did not affect the Th1, Th2, and Th17 cell populations in the PP (Fig. 6a–c). However, BALB/c mice had a lower percentage of FoxP3^+^CD25^-^ Tregs than B6 mice in the PP (*p* < 0.01, Fig. 6d).

**Fig. 7:**
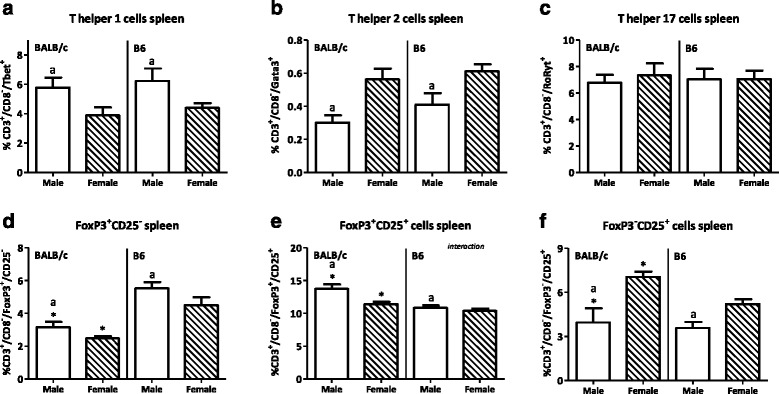
Effect of gender and mouse strain on T helper cell populations in the spleen. Percentage of T helper 1 (**a**), T helper 2 (**b**), T helper 17 (**c**), FoxP3^+^CD25^-^ T regulatory cells (**d**), FoxP3^+^CD25^+^ T regulatory cells (**e**), and FoxP3^-^CD25^+^ effector T cells (**f**) in the spleen of male, female, BALB/c, and B6 mice. T helper cells are expressed as the percentage of cells within the CD3^+^CD8^-^ population. Results are expressed as the mean + SEM and were tested using two-way ANOVA. Significant strain effects are indicated with an *asterisk* (*), and significant gender effects are indicated with the letter *a* in each graph (*p* < 0.05)

In the spleen, males also had a higher percentage of Th1 cells than females (*p* < 0.01, Fig. 7a). While sex did not affect the percentage of splenic Th17 cells (Fig. 7c), the percentage of splenic Th2 cells was reduced in males as compared with females (*p* < 0.05, Fig. 7b). The percentages of both FoxP3^+^CD25^−^ and FoxP3^+^CD25^+^ Tregs in the spleen were higher in males than in females (*p* < 0.05, Fig. 7d, e). However, the percentage of splenic effector Th cells was decreased in males as compared with females (*p* < 0.001, Fig. 7f). Strain had no effect on the percentage of Th1, Th2, or Th17 cells in the spleen (Fig. 7a–c). However, the percentage of FoxP3^+^CD25^−^ Tregs was lower (*p* < 0.05, Fig. 7d), whereas the percentages of FoxP3^+^CD25^+^ Tregs and effector Th cells (*p* < 0.001, Fig. 7e, f) were higher in the spleen of BALB/c mice than of B6 mice. Interaction between the effect of sex and strain was found in the percentage of FoxP3^+^CD25^+^ Tregs in the spleen.

### Effect of sex and strain on innate immune populations in the Peyer’s patches and spleen

#### Dendritic cell subsets

**Fig. 8:**
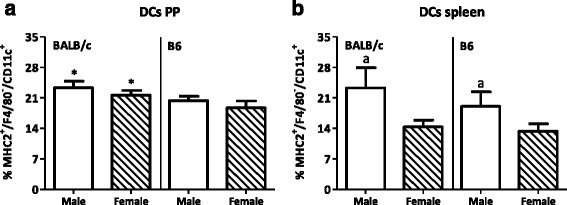
Effect of gender and mouse strain on dendritic cells in the Peyer’s patches and spleen. Percentage of dendritic cells (DCs) in the Peyer’s patches (**a**) and spleen (**b**) of male, female, BALB/c, and B6 mice. DCs are expressed as the percentage of CD11c^+^ cells within the F4/80^−^MHC2^+^ population. Results are expressed as the mean + SEM and were tested using two-way ANOVA. Significant strain effects are indicated with an *asterisk* (*), and significant gender effects are indicated with the letter *a* in each graph (*p* < 0.05)

Sex had no effect on the percentage of DCs (MHC2^+^F4/80^-^CD11c^+^) in the PP (Fig. 8a). However, males had a higher percentage of DCs in their spleens than females (*p* < 0.05, Fig. 8b). BALB/c mice had a higher percentage of DCs than B6 mice in the PP (*p* < 0.05, Fig. 8a). In contrast to the PP, strain did not affect the percentage of DCs in the spleen (Fig. 8b).

**Fig. 9:**
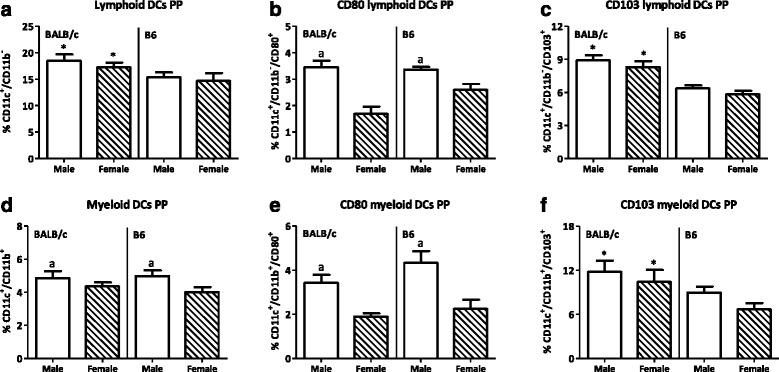
Effect of gender and mouse strain on dendritic cell subsets in the Peyer’s patches. Percentage of lymphoid dendritic cells (**a**) and their expression of CD80 (**b**) and CD103 (**c**) and the percentage of myeloid dendritic cells (**d**) and their expression of CD80 (**e**) and CD103 (**f**) in the Peyer’s patches of male, female, BALB/c, and B6 mice. Lymphoid DCs are expressed as the percentage of CD11b^-^ cells and myeloid DCs as the percentage of CD11b^+^ cells within the CD11c^+^/F4/80^−^MHC2^+^ population. Results are expressed as the mean + SEM and were tested using two-way ANOVA. Significant strain effects are indicated with an *asterisk* (*), and significant gender effects are indicated with the letter *a* in each graph (*p* < 0.05)

In the PP, both lymphoid (CD11b^-^) and myeloid (CD11b^+^) DCs were present. The percentage of lymphoid DCs in the PP was not affected by sex (Fig. 9a). However, males had a higher percentage of CD80^+^ lymphoid DCs in the PP than females (*p* < 0.001, Fig. 9b). Sex did not affect the percentage of CD103^+^ lymphoid DCs (Fig. 9c). Sex had a more pronounced effect on myeloid DCs. Males had an increased percentage of myeloid DCs as compared with females in the PP (*p* < 0.05, Fig. 9d). The percentage of CD80^+^ myeloid DCs was also increased in males as compared with females (*p* < 0.001, Fig. 9e). However, sex had no effect on the percentage of CD103^+^ myeloid DCs in the PP (Fig. 9f). BALB/c mice had a higher percentage of lymphoid DCs than B6 mice (*p* < 0.05, Fig. 9a). Strain did not affect the percentage of CD80^+^ lymphoid DCs (Fig. 9b), whereas BALB/c mice had a higher percentage of CD103^+^ lymphoid DCs than B6 mice in the PP (*p* < 0.001, Fig. 9c). The myeloid DC subset was not affected by strain, with the exception of the percentage of CD103^+^ myeloid DCs in the PP, which was higher in BALB/c mice (*p* < 0.05, Fig. 9f).

**Fig. 10:**
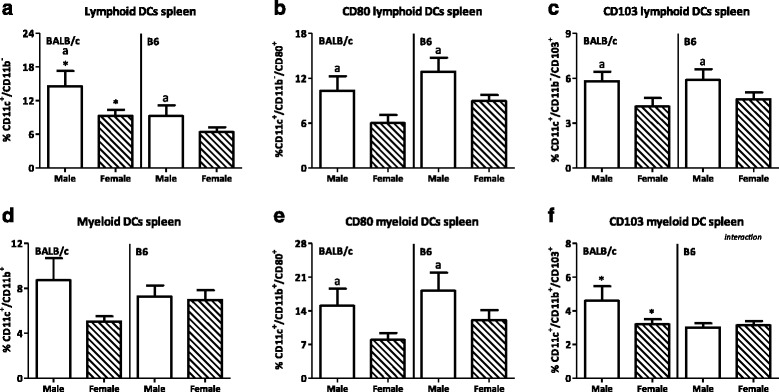
Effect of gender and mouse strain on dendritic cell subsets in the spleen. Percentage of lymphoid dendritic cells (**a**) and their expression of CD80 (**b**) and CD103 (**c**) and the percentage of myeloid dendritic cells (**d**) and their expression of CD80 (**e**) and CD103 (**f**) in the spleen of male, female, BALB/c, and B6 mice. Lymphoid DCs are expressed as the percentage of CD11b^−^ cells and myeloid DCs as the percentage of CD11b^+^ cells within the CD11c^+^/F4/80^−^MHC2^+^ population. Results are expressed as the mean + SEM and were tested using two-way ANOVA. Significant strain effects are indicated with an *asterisk* (*) and significant gender effects are indicated with the letter *a* in each graph (*p* < 0.05)

The effect of sex was also observed in DC subpopulations in the spleen. The lymphoid DC population in the spleen was increased in males (*p* < 0.05, Fig. 10a) as well as the percentage of CD80^+^ lymphoid DCs (*p* < 0.001, Fig. 10b). DCs expressing CD103 were also found in the spleen, though in a lower percentage than the PP. Males had a higher percentage of splenic CD103^+^ lymphoid DCs than females (*p* < 0.05, Fig. 10c). In contrast to the PP, myeloid DCs in the spleen were not affected by sex (Fig. 10d). However, the percentage of CD80^+^ myeloid DCs was higher in males than females (*p* < 0.05, Fig. 10e). Similar as in the PP, sex did not affect the percentage of CD103^+^ myeloid DCs (Fig. 10f). BALB/c mice had a higher percentage of splenic lymphoid DCs than B6 mice (*p* < 0.05, Fig. 10a). However, strain did not affect the percentage of both CD80^+^ or CD103^+^ lymphoid DCs (Fig. 10b, c), the percentage of myeloid DCs or the percentage of myeloid DCs expressing CD80 (Fig. 10d, e). However, BALB/c mice had a higher percentage of CD103^+^ myeloid DCs than B6 mice (*p* < 0.001, Fig. 10f). Interaction between the effect of sex and strain was found in the percentage of CD103^+^ myeloid DCs in the spleen.

#### Macrophages

**Fig. 11:**
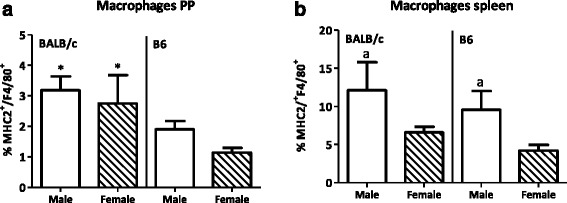
Effect of gender and mouse strain on macrophages in the Peyer’s patches and spleen. Percentage of MHC2^+^F4/80^+^ macrophages in the Peyer’s patches (**a**) and spleen (**b**) of male, female, BALB/c, and B6 mice. Results are expressed as the mean + SEM and were tested using two-way ANOVA. Significant strain effects are indicated with an *asterisk* (*) and significant gender effects are indicated with the letter *a* in each graph (*p* < 0.05)

We only found a small percentage (1–3 % of all leukocytes) of macrophages (MHC2^+^F4/80^+^) in the PP. Sex had no effect on the percentage of macrophages in the PP (Fig. 11a). However, in the spleen, more macrophages were found than in the PP. Males had an increased percentage of macrophages in the spleen as compared with females (*p* < 0.05, Fig. 11b). Strain did affect the percentage of macrophages in the PP. BALB/c mice had an increased percentage of macrophages in the PP as compared with B6 mice (*p* < 0.01, Fig. 11a). However, no effect of strain on the percentage of macrophages was found in the spleen (Figure 11b).

#### Natural killer cells

**Fig. 12:**
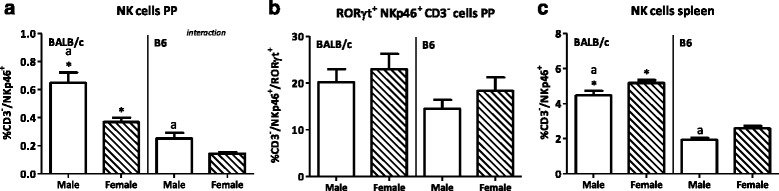
Effect of gender and mouse strain on natural killer cell populations in the Peyer’s patches and spleen. Percentage of NKp46^+^CD3^−^ cells from lymphocytes (**a**), percentage of RORɣt^+^ cells from NKp46^+^CD3^−^ cells (**b**) in the Peyer’s patches, and percentage of NKp46^+^CD3^-^ cells from lymphocytes in the spleen (**c**) of male, female, BALB/c, and B6 mice. Results are expressed as the mean + SEM and were tested using two-way ANOVA. Significant strain effects are indicated with an *asterisk* (*) and significant gender effects are indicated with the letter *a* in each graph (*p* < 0.05)

Only a small percentage of NKp46^+^CD3^-^ (NK cells) were found in the PP, and males had a higher percentage of these cells than females (*p* < 0.001, Fig. 12a). In the PP, two subsets of NKp46^+^ were found, i.e., NKp46^+^RORɣt^+^ and NKp46^+^RORɣt^-^ cells. No sex difference in the RORɣt^+^ cells in the PP was found (Fig. 12b). In the spleen, males had a decreased percentage of NKp46^+^CD3^-^ cells as compared with females (*p* < 0.001, Fig. 12c). Also, strain affected the percentage of NKp46^+^CD3^−^ cells. BALB/c mice had a higher percentage of NKp46^+^CD3^-^ cells in the PP than B6 mice (*p* < 0.001, Fig. 12a). Strain did not affect the NKp46^+^RORɣt^+^cells in the PP (Fig. 12b). Similar observations were done in the spleen. BALB/c mice spleens had a higher percentage of NKp46^+^CD3^-^ cells than B6 mice (*p* < 0.001, Fig. 12c). Interaction between the effect of sex and strain was found in the percentage of NKp46^+^CD3^−^ cells in the PP.

#### Monocytes

**Fig. 13:**
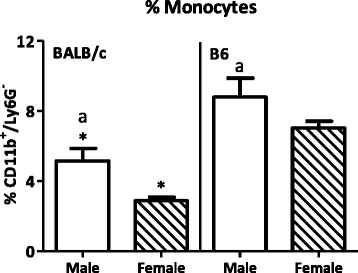
Effect of gender and mouse strain on monocytes in blood. Percentage of CD11b^+^Ly6G^−^ monocytes in blood of male, female, BALB/c, and B6 mice. Monocytes are expressed as the percentage of CD11b^+^Ly6G^−^ cells within the total leukocyte gate. Results are expressed as the mean + SEM and were tested using two-way ANOVA. Significant strain effects are indicated with an *asterisk* (*) and significant gender effects are indicated with the letter *a* in each graph (*p* < 0.05)

Since circulating monocytes are the precursors for DCs and macrophages, we investigated whether the differences in DCs and macrophages, induced by sex or strain, may be related to sex differences in monocyte populations in the blood. Male mice had a higher percentage of total monocytes (CD11b^+^Ly6G^−^) in their blood than female mice (*p* < 0.01, Fig. 13).

**Fig. 14:**
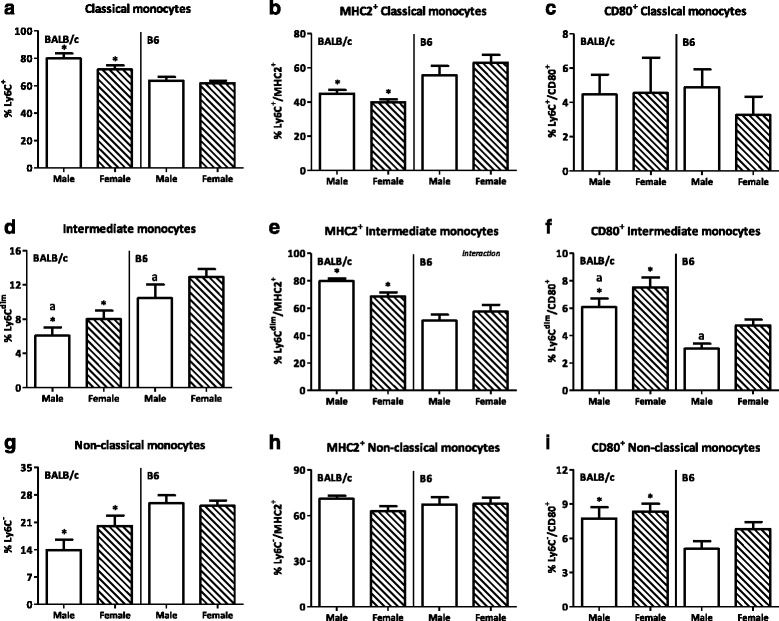
Effect of gender and mouse strain on monocytes subsets in blood. Percentage of classical monocytes (**a**) within the CD11b^+^Ly6G^-^ population and their expression of MHC2 (**b**) and CD80 (**c**). Percentage of intermediate monocytes (**d**) within the CD11b^+^Ly6G^−^ population and their expression of MHC2 (**e**) and CD80 (**f**). Percentage of non-classical monocytes (**g**) within the CD11b^+^Ly6G^−^ population and their expression of MHC2 (**h**) and CD80 (**i**) in blood of male, female, BALB/c, and B6 mice. Results are expressed as the mean + SEM and were tested using two-way ANOVA. Significant strain effects are indicated with an *asterisk* (*) and significant gender effects are indicated with the letter *a* in each graph (*p* < 0.05)

Monocytes can be subdivided into three subpopulations, classical, non-classical, and intermediate monocytes. A trend towards an increased percentage of classical monocytes in males as compared with females was found (*p* = 0.0965, Fig. 14a). Sex did not affect the percentage of classical monocytes expressing the activation markers MHC2 or CD80 [20, 21] (Fig. 14b, c). Sex did affect the percentage of intermediate monocytes (Ly6C^dim^); females had an increased percentage as compared with males (*p* < 0.05, Fig. 14d). However, within this subset, sex did not affect MHC2 expression on intermediate monocytes, whereas the percentage of intermediate monocytes expressing CD80 was lower in males than in females (*p* < 0.01, Fig. 14f). Sex did not affect the percentage of non-classical monocytes (Ly6C^low^) (Fig. 14g) nor the percentage of MHC2^+^ and CD80^+^ non-classical monocytes (Fig. 14h, i). Strain also had an effect on monocytes. The percentage of total monocytes was reduced in BALB/c mice as compared with B6 mice (*p* < 0.001, Fig. 13). The classical monocyte subset was increased in BALB/c mice as compared with B6 mice (*p* < 0.001, Fig. 14a). BALB/c mice had a lower percentage of MHC2^+^ classical monocytes than B6 mice (*p* < 0.001, Fig. 14b), whereas the percentage of CD80^+^ classical monocytes was not affected by strain (Fig. 14c). The percentage of intermediate monocytes was reduced in BALB/c mice as compared to B6 mice, while both the percentages of MHC2^+^ and CD80^+^ intermediate monocytes were higher in BALB/c mice (*p* < 0.01, Fig. 14d-f). The percentage of non-classical monocytes was also reduced in BALB/c mice as compared to B6 mice (*p* < 0.001, Fig. 14g). Strain did not affect the percentage of MHC2^+^ non-classical monocytes, whereas BALB/c mice had a higher percentage of CD80^+^ non-classical monocytes than B6 mice (*p* < 0.01, Fig. 14h, i). Interaction between the effect of sex and strain was found in the percentage of MHC2^+^ intermediate monocytes.

## Discussion

In this study, we show clear sex differences in intestinal and peripheral immune populations. We used two different mouse strains, with different immunological backgrounds and susceptibility to dextran sodium sulfate (DSS)-induced colitis, to validate sex differences. We used the PP as a study site, since it is an important place for immune sampling of antigens from the gut lumen [19]. As a reference for the peripheral immune system, we used the spleen. We focused on Th cell differentiation, DCs, and NK cells because these cells are involved in the maintenance of intestinal immune homeostasis. Just as was reported for systemic differences [3, 4], in general, we found that the innate immune arm (DCs, macrophages, and NK cells) of the PP was enhanced in males as compared with females, as measured by percentage of cells and activation status. The adaptive immune arm in the PP was also different between males and females, since males had a reduced percentage of T cells, while the percentage of Th1 cells within this population was increased. Strain differences were also found in the PP; BALB/c mice had more T cells, a higher Th/Tc ratio, less FoxP3^+^CD25^-^ T cells, more DCs and macrophages, and more NK cells.

Male mice had a reduced percentage of T cells in their PP, although the percentage of Th1 cells within this population was increased. The decreased numbers of T cells in male mice may be due to the higher levels of testosterone, since this hormone may increase T cell apoptosis [22]. Our result is in line with the finding of Giron-Gonzalez et al. (2000), who found an increased peripheral Th1/Th2 cell cytokine profile in men as compared with women [23]. This gender difference may also be due to high levels of testosterone, since testosterone enhances Th1 responses [24]. Despite the fact that both estrogens and testosterone are known to affect Treg cell numbers, we did not find differences in Treg cell numbers in PP between males and females. This appears to be tissue specific, since we did find increased numbers of Tregs in the spleen of male mice.

As the percentage of T cells, which can be influenced by antigen presenting cells, such as DCs and macrophages, were sex dependent, we hypothesized that we would also find sex dependent differences in DCs and macrophages. Indeed, males had an increased percentage of myeloid CD11b^+^ DCs in their PP as compared with females, which were also more mature (increased CD80 expression) [21]. Myeloid DCs are localized in the subepithelial dome (SED) of the PP and were shown to have a particular capacity to produce IL-10 after stimulation and induce the differentiation of Th2 cells [25]. However, these increased myeloid DCs in male mice were not associated with increased numbers of Th2 cells in our study during steady state conditions. In accordance with our finding of no sex differences in the Treg cell populations in the PP, we found no differences in the percentage of regulatory CD103^+^ DCs in the PP, which are able to induce the generation of Treg cells [10]. We found a very low percentage of CD103^+^ dendritic cells in the spleen. Only within the lymphoid DC population, the percentage of CD103^+^ DCs was increased in males as compared with females. In case these CD103^+^ DCs have the same function in the spleen as in the PP, this could indicate that there is an increased potential for more Treg differentiation and a more tolerogenic environment in the spleens of males. This suggestion is in line with the higher percentage of Tregs that we found in the spleens of males than females.

NKp46^+^CD3^-^ cells are also present in the PP and were found to be higher in males than in females. The lower percentage of NK cells in females has been suggested to be caused by increased levels of estrogen [26–28]. Most of these cells are probably conventional NK cells [29]. These more conventional NK cells can secrete, e.g., IFN-ɣ [30], which is an important cytokine for the defense against viral and microbial infections. About 20 % of the NKp46^+^CD3^-^ cells also expressed RORɣt. These cells are thought to be innate lymphoid cells from group three [14, 29, 31] and can produce IL-22 [14]. IL-22 is important in producing antimicrobial products and may protect gut epithelial cells from injury [15]. However, the percentage of these cells were not sex or strain dependent.

As we found sex differences in DCs and macrophages in the PP and spleen, we decided to study circulating monocyte populations as well, since they may be precursors of PP and splenic myeloid DCs and macrophages. Male mice had an increased percentage of total blood monocytes, which is in line with the findings that males have a stronger innate immune response. Mouse monocytes can be subdivided into classical, intermediate, and non-classical monocytes by using the marker Ly6C. Upon entrance in the circulation, monocytes start as classical monocytes with high levels of Ly6C. In the circulation, they maturate into non-classical monocytes, and the expression of Ly6C decreases [32, 33]. Classical monocytes can be recruited to the intestinal mucosa to develop into DCs and macrophages [34]. Within the total monocyte population, we found a trend towards an increased percentage of classical monocytes in males. This may be related to increased numbers of (subpopulations) of DCs and macrophages in the PP and spleen. The increased numbers of classical monocytes in males may also be in line with the increased activation of the innate immune response in males, since it was found that the numbers of classical monocytes increase in inflammatory conditions and infections in mice [35].

Besides sex differences, we also observed strain differences in intestinal immune cell populations in the present study. These differences were expected, since the B6 mice is a typical Th1 responder [36] and sensitive to DSS induced colitis [17, 18], whereas the BALB/c mice is a typical Th2 responder [36] and more resistant to DSS induced colitis [17, 18]. Although there is a lot of knowledge on mouse strain differences in peripheral immunity, our study shows that these immune differences also exist in the PP. We observed that BALB/c mice had more T cells and an increased Th/Tc ratio in both the PP and spleen. In line with our expectation, the colitis resistant BALB/c mice had a higher percentage of FoxP3^+^CD25^+^ Treg cells (measured as percentage from all T cells) in their spleen. This was not found in the PP. However, the percentage of T cells in the PP of BALB/c mice was much higher as compared with B6 mice. This may suggest an increased absolute number of Treg cells in PP of BALB/c mice. Indeed, we found that the percentage of FoxP3^+^CD25^+^ Treg cells from all lymphocytes (instead of from all T cells) was higher in the PP of BALB/c mice than that of B6 mice (data not shown).

The strain effects in the T cell populations in the PP were accompanied with changes in innate immune cell populations in the PP. We observed an increased percentage of total and lymphoid DCs and an increased percentage of regulatory CD103^+^ DCs in the PP of the colitis resistant BALB/c as compared with the colitis sensitive B6 mice. Also, the percentage of macrophages was higher in the PP of BALB/c than B6 mice. Together with an increased percentage of NK cells in the PP, it may be suggested that the innate immune arm in the PP of BALB/c mice is enhanced as compared with the B6 strain. The higher percentage of CD103^+^ DCs in BALB/c mice may be related to the relative resistance of BALB/c mice to colitis induction, since this DC subset is necessary for the induction of colitis protective Tregs [37]. Interestingly, in the peripheral circulation, BALB/c mice had a decreased percentage of monocytes. However, within the monocyte population, these BALB/c mice had an increase percentage of classical monocytes, which is in line with an increased innate immune system in these mice.

In humans, sex differences for several diseases, including IBD, have been described. However, it has also been shown that the sex effects in IBD may be affected by geographical locations, since a female preponderance is found in European and American countries and a male preponderance in Asian countries [5, 6]. This suggests a genetic or environmental influence on sex differences. To test whether sex and genetics can both be involved in IBD susceptibility, we compared males and females of two mouse strains with a different genetic background and a well-known difference in susceptibility for DSS induced colitis [17, 18]. By using a two-way ANOVA, we demonstrated effects of both sex and strain on several immune cell populations, as well as an interaction between sex and strain. This suggests indeed that both sex and genetics are involved in immune cell differences in mice and that genetics influenced the sex-induced immune differences in mice. The sex-dependent differences in IBD susceptibility might be explained by the increased percentage of Th1 cells, i.e., IFN-γ producing T cells, in the PP of males, since Th1 cells and IFN-γ are associated with the development of colitis [38]. Also, the increased percentage of NK cells in males might contribute to the increased development of colitis in males [39]. Another contributing factor might be the higher percentage of mature CD80 expressing DCs in the PP of males, as these cells are more capable of activating T cells and inducing immune responses as compared with immature DCs [21, 40].

To the best of our knowledge, this is the first study showing sex differences in intestinal immune cell populations in mice. In order to gain more insight in the implications of these sex differences, future studies are required. This could include studies investigating the effect of sex on intestinal immune populations in challenge models like colitis (IBD) or oral infections, such as salmonella. Salmonella would be interesting, since it can enter the body via the PP [41]. Moreover, several other mechanisms may be involved in the pathogenesis of intestinal diseases, such as changes in the intestinal microbiota composition [42, 43] or decreased mucus production in the intestines [44]. Therefore, future studies should also focus on the effect of sex on these mechanisms, as well as on the relation of these mechanisms with the intestinal immune response. Finally, the role of sex hormones in the induction of the sex differences should also be studied.

## Conclusions

This study showed for the first time sex differences in intestinal immune populations in mice. The innate immune arm (DCs, macrophages, and NK cells) of the PP appeared to be enhanced in male versus female mice (as judged by the number of cells and their activation status). On the other hand, the adaptive immune arm seemed to be decreased in the PP of males, since they had a reduced percentage of T cells, although the Th1 cells within this population were increased. The sex differences we found may underlie the sex differences in intestinal disorders, like IBD. Furthermore, this information may be an important knowledge not only for the treatment of intestinal related diseases but also for the development of functional foods, like pre- and probiotics.
